# The Impact of Translational Neuroscience on Revisiting Psychiatric Diagnosis: State of the Art and Conceptual Analysis

**DOI:** 10.4274/balkanmedj.2017.1190

**Published:** 2017-12-01

**Authors:** Massimiliano Aragona

**Affiliations:** 1 Crossing Dialogues Association, Rome, Italy

**Keywords:** Translational research, psychiatric classification, psychopathology, validity, Research Domain Criteria, Cambridge model

## Abstract

This paper reviews translational research in psychiatry, focusing on those programs addressing the problem of the validity of psychiatric diagnoses. In medicine in general, and in psychiatry in particular, the term “translational” is used with different meanings. A conceptual analysis suggests that there are at least seven different types of translational research in psychiatry: T1 (“bench-to-bedside” development of tools and treatments), T2 (application of animal models to human psychiatry), T3 (papers focusing on the mind-brain gap, studying biological, neurobiological and cognitive dysfunctions), T4 (personalised therapies and prediction of treatment responses), T5 (“bedside-to-bench” translation of population data for laboratories), T6 (implementation of treatments at the population level, including accessibility and quality of services), and T7 (improving translational knowledge in residents’ trainings and researchers’ careers).

Concerning the problem of validity of psychiatric diagnoses, new neurocognitive models like the Research Domain Criteria project are considered, in particular the translational program of cross-validation aimed at reducing the gap between neuroimaging data and psychopathological scores derived from rating-scales. It is shown that these programs are useful, filling some of the current research gaps, but it is also stressed that they carry implicit realist and reductionist assumptions. It is finally suggested that the formation of mental symptoms is a complex process involving both neurocognitive and semantic factors, which raises doubts about the possibility of complete translations, without residuals.

Once upon a time there was a psychiatric community believing in the “bible” of psychiatry (the DSM, Diagnostic and Statistical Manual of Mental Disorders). There were rigorous operational criteria functioning as mechanisms in which the input was the symptoms found by the clinician and the output a diagnostic label. Such diagnoses, called mental disorders, were assumed to be natural entities like many other medical diseases, the only problem being that their aetiopathogenesis was still unknown. However, there was a general trust that with time researchers would have discovered the specific genetic liabilities, neurochemical imbalances and neurocognitive circuits responsible for mental disorders.

Many were surprised to realise that the long awaited DSM-5 (1) was published in the midst of unusual controversy, with the credibility of the “bible” being put in question. For example, the former president of the World Psychiatric Association, Maj (2), commented that since the publication of the DSM-IV: “Only a couple of decades have passed, but those already seem “good old days”. Much of that enthusiasm and faith has now vanished […] the questions I am now receiving from journalists […] focus not so much on “new developments in the manual” (the most common question when the DSM-IV was launched) as on […] “why we produce this classification at all, since we do not have a solid ground on which to base it”. What had happened? Was it predictable?

An epistemological analysis inspired by the work of Kuhn (3) had been proposed years before to highlight the structural problems responsible for many “empirical” difficulties encountered when the diagnostic criteria of the DSM-3 (and later editions) had been applied in clinical and research settings: internal heterogeneity of the diagnostic categories, excessively high rates of comorbidity, lack of prognostic and treatment specificity, questionable validity, and so on (4,5). The main thesis was that such “empirical” difficulties were instances of Kuhnian “anomalies”, i.e. apparently empirical outputs which largely depended on the way in which the system was internally structured. As a consequence, it was the structure of the DSM itself that was under pressure, and a possible “revolutionary” system was awaited.

In the meantime, research in neuroscience was dramatically progressing thanks to the availability of new neuroimaging and neurophysiological techniques. However, the consequent better knowledge of brain structure and function raised new challenges when applied to psychopathology. On the one hand, there are projects of radical reformulation of psychiatry based on the neurosciences (6,7,8), while on the other hand there are several authors suggesting that their time has not yet come (9,10,11).

The pressing question raised by these new developments is whether mental symptoms are reducible to neurocognitive function without residual, i.e. how much the capture of brain dysfunction is related to the psychopathological phenomena that are supposed to arise from it? It is in this context that the problem of “filling the gap” between phenomena and brain activity arises anew.

Recently, the term “translational” has been imported into psychiatry to refer to research aimed at resolving this problem. However, as we will see, there are several meanings attached to this word, hence some preliminary conceptual clarification is needed.

In this paper, I will first review the meanings of “translational research” in medicine. Second, I will perform a PubMed review of translational research in psychiatry to show its already established uses and main tendencies. Finally, I will focus on translational research applied to the problem of validity of mental disorders in relation to neuroscience.

## Translational research in medicine

According to Woolf (12), “Translational research means different things to different people, but it seems important to almost everyone”, although these differences may engender some confusion. In general, it is part of translational research whatever “seeks to ‘translate’ research in ways that enable that research to be applied” (13), and classically there are two major meanings, i.e. T1 and T2. T1 refers to the so-called “bench-to-bedside” process of harnessing knowledge from basic sciences to produce new drugs or instruments for treatment. Its primary endpoint is the production of new treatments that can be “brought to market”; its main instruments are clinical trials designed to test possible application to humans of the results of laboratory research and preclinical studies. T2 refers to the concrete application of the products of T1 to daily clinical activity, translating research into practice by enhancing the adoption of best practices. According to Woolf (12), T1 research requires mastery of molecular biology, genetics, and other basic sciences, strong laboratories and cutting-edge technology, while the “laboratory” for T2 research is the community and ambulatory care settings, where this second kind of translational research focuses on improving access to services and quality of assistance. To this distinction, Westfall et al. (14) added a third step (T3) called “practice-based research”, aimed at facilitating the implementation of systematic reviews and guidelines into clinical practice. More recently, Mitchell (13) itemised translational research in a rational order from basic to general applications, i.e. from T0, fundamental research discoveries, to T5, implementation of population findings at global level (Table 1).

As we will see, in psychiatry, “translational research” is used in different ways that only partially overlap with the medical usage, so a different list of “T” types will emerge.

## Translational research in psychiatry: a review

**FIG. 1.**
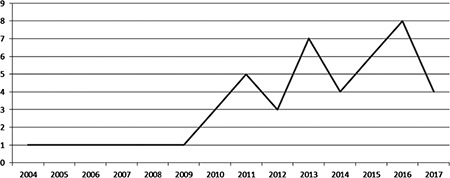
Number of publications on translational research in psychiatry, per year.

A PubMed search (performed August 30th, 2017) revealed 50 articles containing “translational research” AND “psychiatry” either in the title, keywords or abstract. Of these, 3 did not focus on psychiatry and 1 was a historical paper on asylums. Of the remaining 46 papers, 20 (43.48%) were reviews, 16 (34.78%) were theoretical papers, 4 (8.70%) were abstracts or reports of meetings, workshops or roundtables at congresses, 2 (4.35%) were commentaries and 2 (4.35%) were experimental studies: one studied the interplay between the workflow for clinical tasks and research data collection (15), and the other measured sensitivity and specificity of a vector machine model for the analysis of transcriptomic data in major depressive disorder (16). Finally, there was one editorial and one paper describing a device (the development of a database). As shown in Figure 1, the publication of studies on translational research in psychiatry is recent; the papers retrieved in our review started from 2004, with an increase in number of publications in the most recent years. Of the 46 articles considered, 35 (76.09%) were focused on translational research issues, while translational research was not the major theme in 11 papers (23.91%) where it was just mentioned (for example as a future development). The majority of studies were on psychiatric or neuropsychiatric disorders in general, schizophrenia, depression and autism.

The analysis of the typology of translational research (T types) presented in these papers was performed with a bottom-up procedure, starting from major concepts similar to the T1/T2 distinction used in medicine, and adding or dividing such categories when new stances emerged. It is presented in detail in Table 2. In one case it was difficult to decide the T type. The remaining 45 articles were divided into the following T types:

T1 (n=15 articles, 33.33%) includes “bench-to-bedside” papers, i.e. basic science developments of tools and treatments that could be used clinically or commercialised.

T2 (n=5, 11.11%) comprises articles arguing for the application of animal models to human psychiatry.

T3 (n=7, 15.56%) contains papers focusing on the mind-brain gap, i.e. focusing on aetiology. pathophysiology and/or validation of mental phenomena/disorders through neurobiological research or the study of neurocognitive dysfunctions.

T4 (n=3, 6.67%) includes articles about personalised therapies and the possibility to predict/track treatment response.

T5 is a category with one article proposing a reverted “bedside-to-bench” project aimed at translating knowledge generated at population level to laboratories.

T6 (n=10, 22.22%) is aimed at translating research into practice (improving the implementation of treatments, accessibility and quality of services, and so on).

T7 (n=4, 8.89%) comprises papers proposing ways to improve translational knowledge in residents’ trainings and researchers’ careers.

In general, in psychiatry translational research appears to be a recent movement that has been rapidly growing in most recent years. However, absolute numbers of publications are still low, in one third of the papers translational research is not the main issue and, above all, the vast majority of articles are not experimental. Moreover, the analysis of T types shows a relevant heterogeneity, with at least seven different kinds of translational research.

In the next section, I will explore in more detail the T3 typology, dealing with the impact of translational neuroscience, particularly in validating/revising psychiatric diagnoses.

## Neurocognitive sciences and psychiatry

“Translational research” is just one of several terms used to refer to three related questions: a) what are the neurocognitive dysfunctions subtending mental illness? b) what are the research methods to fill the gap between “objective” neuroscientific data and “subjective” mental phenomena? c) what is the importance of these studies in revising psychiatric classification? Among the terms used to refer to these issues, the neurophenomenological movement and the work on endophenotypes would deserve thorough consideration, although for reasons of space they will not be considered here. More recently, the raise of neurocognitive research fuelled programs of neurocognitive reformulation of psychiatric research and classification, the most known being the Research Domain Criteria (RDoC) project (8). Quite surprisingly, only one of the articles found in our review dealt with the RDoC project, discussing the application of an RDoC framework to research into maltreated children (17). Among the articles pertaining to T3, one proposes a multi-dimensional translational research focusing on neuron-glia interaction as a possible glue to reduce the mind-brain gap (18). Another article studies a neurophysiological index, the pre-pulse inhibition of startle response, whose impairment has been reported in several psychiatric diseases, particularly schizophrenia (19). Schizophrenia is also the topic of a systematic review on the contribution of resting-state Magnetoencephalography in elucidating abnormal neural organisation in schizophrenic patients (20). Two articles focus on the specificities of geriatric depression, one considering the dysexecutive syndrome typical of the aging-brain as a key to the neuropsychology of geriatric depression (21), and the other dealing with the development and application of integrative approaches combining basic research (genetic) and clinical neuroscience (neuroimaging) to study treatment response variability, medical comorbidity, and the potential overlap between depression and dementia (22). Moreover, Sharp et al. (23) propose a “multilevel research approach” that combines performance on behavioural economic experiments (reward-related decision-making) with brain activity at neuroimaging. Finally, Khalsa and Lapidus (24) propose “interoception” as a viable possibility for translational research linking neural bases, measurable biomarkers and transdiagnostic psychiatric targets.

Returning to the RDoC project, it was designed “to shift researchers away from focusing on the traditional diagnostic categories as an organising principle for selecting study populations towards a focus on dysregulated neurobiological systems” (25). Although epistemologically there are several limitations of the RDoC project, it is predictable that “in the next years it will become a must do for many new research projects” (26). Recently, it was suggested to revise psychiatric classification according to the RDoC model, together with the introduction of translational validation (27). This contribution to the debate does not always find space in journals indexed in PubMed, so it was not included in the review presented above. However, considering that it focuses specifically on our main theme (i.e. translational research and psychiatric validation), it deserves to be discussed. In an earlier paper, Stoyanov (28) proposed a “validation-theory” that, starting from existing discrepancies between the methods of neuroscience and those of psychopathology, aimed at exploring and “cross-validating” neuroimaging data and answering in clinical rating-scales. In his view, neuroscientific results can be a possible source of external validity for psychiatric conditions, but current research procedures do not introduce relevant rules for translation of the data among these interconnected domains (29). In particular, one problem would be the temporal gap between the administration of self-rating instruments and the following brain-scanning procedures, a gap that loosens the correlation and makes it difficult to interpret neuroimaging results as related to the psychopathological scores. More recently, this view received initial empirical support from a study (30) testing the feasibility of real-time administration of clinical rating scales during fMRI scanning (simultaneous administrations) in depressed patients compared to healthy volunteers. This study showed selective correspondence between depressive responses and brain activations in areas believed to be implicated in the pathophysiology of depressive conditions. Although preliminary, these results confirm that the temporal gap of administration can be overcome.

## DISCUSSION

This paper reviewed translational research in psychiatry, focusing on those programs addressing the problem of the validity of psychiatric diagnoses.

In general, translational research in psychiatry appeared quite heterogeneous, with at least seven different ways to intend such programs. This polysemy may engender confusion and a first part of this paper was dedicated to a conceptual clarification to reduce misunderstanding.

Another problem is that the debate on the potential of neurocognitive research in revisiting psychiatric diagnoses is only discussed in part within the framework of translational research; several other terms and concepts are involved as well.

One major issue in contemporary psychiatry is the validity of psychiatric diagnoses, because the crisis of confidence that emerged in recent years in relation to the DSM-5 (31) is not only a nosological problem but a wider one, challenging psychopathological research as a whole (32).

In general, psychiatry is at a crossroads. On the one hand, there is the classic psychiatric stance, whose last version was the neo-Kraepelinian project. According to this perspective, psychiatrists had to reliably describe mental disorders, i.e. phenomenally-based clinical descriptions of conditions that were considered putative disease entities. Subsequent research should have gone through validation research to discover the neurobiology subtending such conditions. On the other hand, following the advancements of recent neurocognitive science, new proposals flourished suggesting the need to consider neurocognitive dysfunctions as the key elements to be studied, with psychopathological phenomena (mental symptoms) secondarily arising from this more basic level.

In other words, while in the neo-Kraepelinian approach validation research is expected to proceed from phenomenally defined disorders back to the discovery of their aetiology, in programs like the RDoC project, the direction is expected to be from “subpersonal” dysfunctions (of genes, brain processes, or cognitive mechanisms) ahead to the resulting phenomenal picture (33). Both views have a similar reductionist stance, because they see mental pathologies as biomedical entities resulting from a dysfunction of physiological processes; the difference is just in the direction of the discovery enterprise (from phenomena to pathophysiology or vice versa).

According to Telles Correia (34), both approaches pertain to a “realistic view” in which validity is seen as corresponding to reality “as it is”, while other views may consider mental disorders as practical kinds and validity as adequacy to the reality as we see it and how we deal with it. I guess that the former stance is often the one that neuroscientists have in mind when they propose revising psychiatric diagnoses in neurocognitive terms. However, in my opinion, translational research trying to find better ways to correlate mental phenomena and neurocognitive functioning does not need to be necessarily reductionist. The main problem should not be how to substitute subjective complaints with objective neurocognitive data, because both are proxies. Scores on rating-scales are proxies of the lived experiences and behaviours of the patients, and the path from self-experience to clinical “objectification” may be long and require self-interpretation (by the patient), understanding of the items’ content and (in case of clinician administered rating scales) interpretation by the clinician. Colours of neuroimages are proxies of particular targets (e.g. bold signal) which in turn are proxies of supposed cognitive functions. Accordingly, the main issue should be how to improve the capture of these proxies in order to reduce the slippage of information that currently exists between the phenomenal level and the neurobiological one (35).

As a result, one main issue for psychopathological research is the following: researchers working in translational research applied to the validity of psychiatric diagnoses should consider that the relationship between neurophysiological variables and mental symptoms is much less direct and linear than commonly claimed. For example, the Cambridge model of symptom formation suggests that mental symptoms may be conceived as complexes of neurobiological and “semantic” (individual, socio-cultural and dialogical) elements. According to this concept, even when a neurobiological signal is the starting point of the constructive process, the degree of its correspondence to the final mental symptom is variable (36) and depends on the degree of involvement of semantic modulatory factors on the original brain signal. Accordingly, “the fewer modulatory factors involved, the closer the final symptom will be to the original brain signal. The more modulatory factors, the less representative it will be to the point that nothing in the final symptom will be redolent of its original brain address” (37).

Bearing in mind this cautionary knowledge does not mean that translational research pointing to better ways to correlate mental and neurocognitive phenomena should be viewed with suspicion. On the contrary, such attempts to better articulate the relationship between the phenomenal and the neurocognitive level should be welcomed, provided that translation should be allowed in both directions and that any good translator has to be aware that “to translate” is also, a little bit but unavoidably, “to betray”.

## Figures and Tables

**Table 1:**
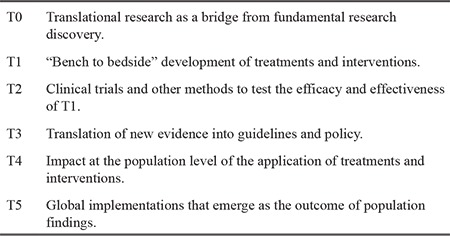
Typologies of medical translational research (13)

**Table 2:**
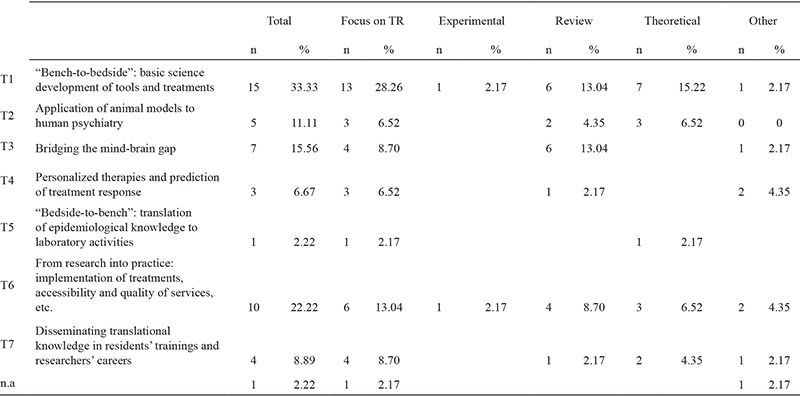
Typologies of psychiatric translational research, rates of articles focused on it, and kind of publication
